# Mixed effects of protected areas on avian food webs

**DOI:** 10.1098/rspb.2025.0614

**Published:** 2025-06-04

**Authors:** Lucie Thompson, Nuria Galiana, Konstans Wells, Miguel Lurgi

**Affiliations:** ^1^ Department of Biosciences, Swansea University, Swansea, UK; ^2^ Museo Nacional de Ciencias Naturales, Madrid, Spain

**Keywords:** ecological networks, trophic interactions, habitat degradation, biodiversity conservation, protected areas, food webs, biogeography

## Abstract

Protecting habitat and the species they shelter by setting up protected areas (PAs) has become a conservation priority to mitigate the current extinction crisis. This strategy has improved different aspects of biodiversity including species richness and abundance across ecosystems. However, to truly understand the effectiveness of mitigation measures against global environmental change, we must account for one of the fundamental dimensions of biodiversity: species interactions. Using 376 556 curated citizen science records of 509 bird species distributed across 45 networks of European PAs (collections of connected PAs), we show that the effects of PAs on the structure of avian food webs are mixed across Europe. Overall effects of protection include an increase in species richness and larger body masses of both top and intermediate species. For other food web features, the sign and magnitude of the effects are mixed. Our results further suggest that these effects are strongly influenced by geographical and environmental features of the PA networks such as remoteness, habitat diversity, human pressure and agriculture. Lastly, PAs with specific protection goals such as those administered by European Bird Directives for conservation sustain more complex food webs. Our study provides evidence for the need of including clear management goals and considering environmental context in the designation of PAs to increase their effectiveness at preserving biodiversity beyond species richness.

## 1. Introduction

With the expansion of human populations around twelve thousand years ago, the Earth started to transition into a period that we now know as the Anthropocene. Owing to anthropogenic change, our world has, since then, experienced hundreds of species extinctions and range contractions [1,2]. This has resulted in a global collapse of biodiversity and species abundance, with fundamental repercussions for the insidious extinctions of ecological interactions that glue communities together [1,3]. The selective loss of species with particular traits such as large species [4–7], mammals [8] and top predators [9] has caused a fundamental rearrangement of natural communities and food webs [8,10].

Protected areas (PAs), by setting aside natural areas and shielding them from some or all human pressure [11,12], represent the main conservation strategy to slowing down the biodiversity crisis. They have been proven effective at slowing down species, functional and phylogenetic diversity loss, as well as population declines [13–18]. Nonetheless, if we are to fully understand the effects of anthropogenic changes on biodiversity and therefore the effectiveness of PAs at ameliorating them, we need to consider their influence on species interactions [19,20]. Ecological communities are more than the sum of their parts. They are complex systems of interacting species that create emergent patterns and functions [5,21]. Considering them in biodiversity surveys is especially important since ecological interactions can be lost even before the species involved go extinct [3,22]. In addition, many food web metrics are believed to affect the stability and resilience of communities to disturbances [23–27] and their cascading effects [28,29]. They can inform on the amount of energy necessary to sustain predators [30,31], but also can also constitute vectors of disturbance transmission [32].

Thus, the structure of biotic interactions can inform on a community’s ability to withstand anthropogenic perturbations. As such key drivers of community integrity and provision of crucial ecosystem services [33], systematic inclusion of biotic interactions into biodiversity assessments might provide important insight for conservation actions [19,20].

We already know that gradients of anthropogenic disturbance negatively impact the structure of food webs. For example, species richness, the average length of food chains and the fraction of omnivorous species decrease along gradients of habitat degradation [34–36]. Concomitant consequences of these changes on food webs include a reduction in their modularity, owing to a relative increase in trophic interactions when species are lost ([35,37] but see [28,38]), with consequences on stability through the transmission of disturbance between species and modules. Food webs also become shorter and less pyramidal, shifting towards a ‘flatter’ structure with more, but smaller, top predator species owing to the loss of the more vulnerable, larger species [4–7,38,39]. Although species-focused assessments of PA efficacy have provided useful insight into the effectiveness of PAs at protecting specific species, species groups and species diversity [15,17,40], it remains unknown to what extent are PAs able to preserve the structure of species interactions networks. Here we ask three fundamental questions: first, whether PAs have an effect on food web structure compared to non-protected food webs in their immediate vicinity; second, which food web features are most affected by PAs; and third, what are the drivers of PA effect on food webs across PA networks.

We assessed the ability of PAs to mitigate the impacts of ecosystem degradation on food webs across Europe by combining two large global-citizen-science databases of species occurrences, Global Biodiversity Information Facility (GBIF) [41] and eBird [42], with a comprehensive, taxonomically resolved food web of tetrapod species across Europe [43]. From 376 556 individual records of 509 bird species across 45 networks of PAs and surrounding areas in Europe, we built local avian food webs at the 10 × 10 km cell level and computed 16 food web metrics to quantify their structure and composition. By contrasting these metrics between PAs and non-protected surrounding areas, we examined the effects of protection on food webs. Furthermore, we investigated how different features of PAs and their geographical and environmental context influence the outcome of protection.

## 2. Methods

### (a) Species occurrences

We focused our study on species included in the tetrapod European food web Tetra-EU [43], a species-level trophic (i.e. comprising feeding interactions) metaweb comprising 1151 tetrapod species and 60 435 trophic interactions. To our knowledge, it is the only well-resolved continental-scale ecological network characterized to date. This limited the geographical scope of our study to the European continent. We built local communities from species occurrence data downloaded from two citizen science projects: the GBIF [41] (two-thirds of records) and eBird [42] (one-third of records). These data were filtered for observations of birds included in Tetra-EU [43] during the breeding period (April–August) between 2000 and 2022 and within our study area (electronic supplementary material, figure S1). See the electronic supplementary material, Methods for more detail on filtering protocols and taxonomic homogenization. Filtering resulted in 509 bird species considered for analysis.

**Figure 1.**
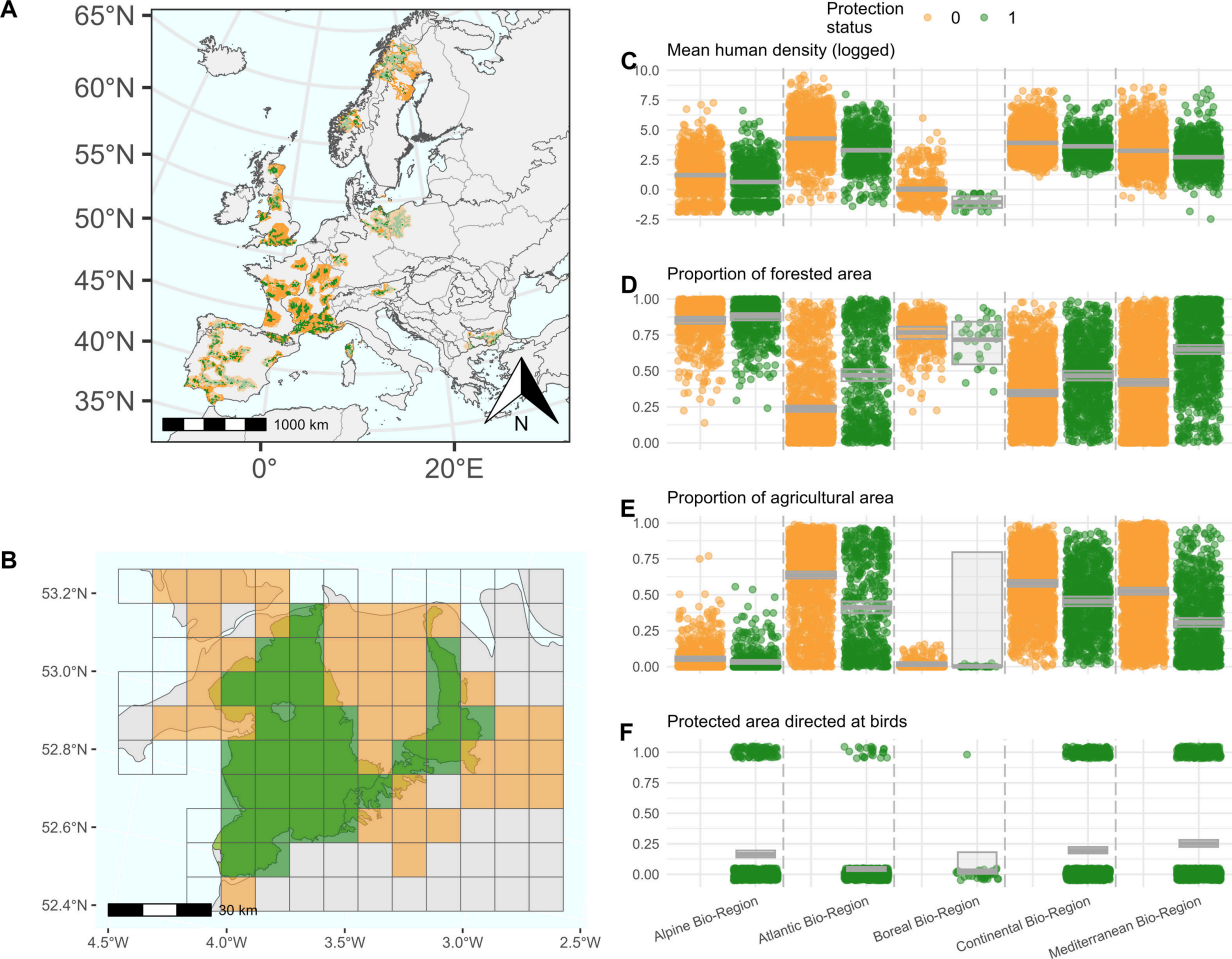
Location of PA networks studied across Europe and corresponding levels of anthropogenic disturbance. The map in (A) shows the 45 PA networks studied. Each PA network represents a grouping of connected PAs (less than 1 km apart) within the same biogeographical region and established before 2015. Opacity of the grid cells distinguishes cells used for quantifying food web metrics; darker—electronic supplementary material, Data 1) and those only used to describe the environmental characteristics of the PA networks (whole dataset—8932 grid cells; lighter—electronic supplementary material, Data 2). Lighter grid cells were left out of the biodiversity analyses owing to low sampling effort. Non-protected (orange) grid cells lie within 100 km of the protected (green) ones. Grey boundaries represent country boundaries and black boundaries represent biogeographical region boundaries. (B) shows a sample PA network in Wales, United Kingdom. Only coloured grid cells were used for analysis (sufficient survey effort), but all grid cells (coloured and transparent) were used to evaluate environmental characteristics of the PA network. (C–E) Graphs contextualize our 45 PA networks in terms of their anthropogenic pressures. They illustrate mean human density, fraction of forested or agricultural areas in protected and non-protected land across each of the five bioregions represented in our study. (F) shows the fraction of PAs especially designated for the protection of bird species within our PA networks. Horizontal bars and shaded area on the data distributions in (C–F) represent the estimate and 95% confidence intervals around the effect of protection status on the environmental characteristic using linear (C) or logistic regressions (D–F).

### (b) Reducing biases in sampling effort

We divided Europe into 10 × 10 km grid cells (electronic supplementary material, figure S1). We calculated sampling effort as the number of survey events per grid cell between 2000 and 2022. One survey event was defined as a unique survey visit to a location: the combination of date, time, location and survey programme from which the record originated (e.g. INaturalist, observation.org, eBird). To avoid potential sampling biases, we dealt with uneven sample size by first removing from our analyses all grid cells with a number of survey events smaller than 50. Second, we rarefied the number of survey event $N_{z,i}$ for grid cell *z* in PA network *i* to that of the grid cell with the smallest number of survey events $N{min}_{i}$ in PA network *i*. This was done by randomly drawing without replacement $N{min}_{i}$ survey events (survey programme × date × time) among all survey events $N_{z,i}$, for which we then extracted the rarefied species occurrence records. This rarefaction ensures constant survey effort across all grid cells within a PA network and reduces chances of rare species being over-represented in intensively sampled sites.

Environmental characteristics of PA networks (see ‘Statistical analysis’ below) were based on all grid cells (regardless of sample size). Cells with at least 50 survey events were used to compute food web metrics (see ‘Local food web construction’ below). In the whole dataset (transparent grid cells in figure 1), PA networks had on average 66 protected grid cells (min = 16, max = 397) and 132 non-protected grid cells (min = 20, max = 543). In the subset dataset used for statistical analysis (opaque cells in figure 1), PA networks had on average 31 protected grid cells (min = 15, max = 99) and 70 non-protected grid cells (min = 19, max = 348).

### (c) Local food web construction and metrics

We built food webs at the local scale of the 10 × 10 km grid cell by determining pairwise co-occurrence of species within a cell and assuming that a trophic interaction was present in the web if the co-occurring species are involved in a trophic interaction in the European Tetra-EU metaweb [43]. Thus, we assumed that an interaction between two species was realized within local communities whenever they co-occurred in a grid cell and an interaction between them has been reported at the European scale.

Thirteen food web metrics and three body size metrics were calculated on the food webs constructed locally at each grid cell (dataset with food web metrics can be found in the electronic supplementary material, Data 1). These included measurements of:

(i) food web topology: (1) modularity, (2) mean food chain length (MFCL), (3) mean trophic level and (4) fraction of omnivory;
(ii) species’ diet composition: (5) mean generality (number of prey items) and (6) mean vulnerability (number of predators);
(iii) food web complexity: (7) species richness, (8) number of links, (9) fraction of realized links and (10) number of links per species;
(iv) species composition: number of (11) basal, (12) intermediate and (13) top species; and
(v) species’ traits: (14) basal, (15) intermediate and (16) top species’ body size.

More details on the definition and computation of those metrics can be found in the electronic supplementary material, table S2. Given that our study is concerned only with birds, the notion of basal species refers to birds that do not feed on other birds (insectivorous, granivorous, etc.) as opposed to the ‘classic’ idea of a basal species that are usually either herbivores or primary producers.

### (d) Protected areas

The data on PAs were extracted from the World Database of Protected Areas (WDPA) [44]. The WDPA was processed by removing UNESCO world heritage sites and maritime PAs, transforming all multi-polygons into simple polygons and removing polygons with an area smaller than our grain size—10 km² (as in [45]) and PAs designated after 2015. These recently established PAs were removed because we considered that not enough time has passed for the food webs inside them to be affected by protection status.

### (e) Study design

#### (i) Comparison of protected and surrounding non-protected grid cells

Each grid cell was assigned one (or several) protection status by intersecting the WDPA shapefile with the European grid. To maximize sample size, grid cells were considered protected if their centre overlapped with a PA polygon, otherwise they were considered non-protected. For grid cells intersecting with several overlapping PAs, the grid cell was assigned the higher protection level (scored from 1 to 10, from no status recorded, regional parks to national park or strict nature reserves). For overlapping PAs with identical protection levels, we filtered by age, keeping the oldest. Finally for PAs with identical age and protection level, we kept the largest. For all the remaining overlapping PAs, we randomly assigned one PA to the grid cell.

#### (ii) Grouping protected area into protected area networks

To account for the fact that, in Europe, PAs are often clustered and small, we proceeded to group ‘connected’ PAs (i.e. within 1 km of each other). This grouping allowed us to create larger areas, thus increasing sample size. In addition, we deemed this method more realistic in terms of the scale at which birds tend to move, as well as a way to account for the complementarity and non-independence of different types of closely situated PAs [46].

To compare protected grid cells to their non-protected counterparts, we restricted our comparison to the cells within 100 km of a PA and in the same bio-geographical region (see below). A set of protected and its non-protected grid cells is hereafter called a ‘PA network’.

Our use of a 100 km buffer zone is a large area to consider as comparison. This large number was chosen mostly to increase our sample size of PA networks considered. In practise, however, PAs being so clustered in Europe, 75% of our PA networks were compared with non-protected communities within 55 km of their boundaries.

For each PA network, we kept only occurrence records that were recorded after the designation of the youngest PA within each PA network. For example, if the PA network is constituted of 10 PAs among which the youngest PA was designated in 2010, we keep occurrence records from 2010 onwards (12 years of records) for the whole PA network—to ensure that all our local communities were protected at the time when their bird population was surveyed. In addition, we only kept PA networks with at least 15 protected grid cells and 15 non-protected grid cells yielding a total of 46 PA networks.

From these 46 PA networks, one very large PA network situated in Poland had very few sampled grid cells relative to its total area (a ratio of 3% of the grid cells were ‘correctly’ sampled, all other PA networks being between 12 and 97%). Thus, we deemed this PA network to be poorly represented by our occurrence data and removed it from all subsequent analyses. Thus, our analysis was conducted on 4591 grid cells spread across 45 PA networks distributed mostly in western Europe with some PA networks also in Germany, Poland, Austria, Norway, Sweden and Bulgaria (figure 1). The average PA network was made of seven (s.d. = 6, min = 1, max = 123) PAs.

### (f) Statistical analyses

#### (i) Difference in food web structure between protected and non-protected grid cells

For each PA network, we quantified the relative strength $\beta_{i}$ of differences in food web metrics between protected and non-protected food webs. This was conducted using a generalized additive mixed model (GAMM) (*mgcv* package [47]):


(2.1) $$\begin{array}{l} food\;web\;metric∼\beta_{0}+\beta *protection\;status+s\left(survey\;effort\right)+s\left(species\;richness\right)+\;\left(elevation\;|\;PA\;network\right)+\left(remoteness|PA\;network\right)+\left(\;1|\;land\;cover\;type\right)+\;\beta_{i}*\;\left(protection\;status\;|PA\;network\right)+\left(1|PA\;network\right). \end{array}$$

Here, the β coefficient equation (2.1) estimates the overall expected differences in the corresponding food web metric in relation to protection status, whereas the parameters of interest are the random effect coefficients $\beta_{i}$, which (according to the variable-slope model structure) return for each PA network the site-specific estimate of the inside-outside difference in food web metrics.

Within this model, the random slopes (elevation | PA network) and (remoteness|PA network) allowed us to control for the bias in elevation and slope within PA networks. Indeed, PA locations are known to be biased towards cells that are ‘unlikely to face land conversion pressures even in the absence of protection’, i.e. at higher elevations, steeper slopes and greater distances to roads and cities [48]. We also controlled for differences in sampling effort (before rarefaction), species richness and land cover across grid cells as those are global drivers of food web structure [49]. These were included as a random effect for land cover type (CORINE Landcover [50]) and nonlinear smoothing terms for survey effort and species richness (for all food web metrics except species richness for the latter). Land cover types included artificial surfaces, agricultural areas, forest and seminatural areas, wetlands and water bodies.

Different error distributions were used depending on the nature of the food web metric (dependent variable): count metrics were modelled using a negative binomial error distribution, proportion data using quasi-binomial error distribution with a log link and continuous metrics using a Gaussian distribution.

**Figure 2.**
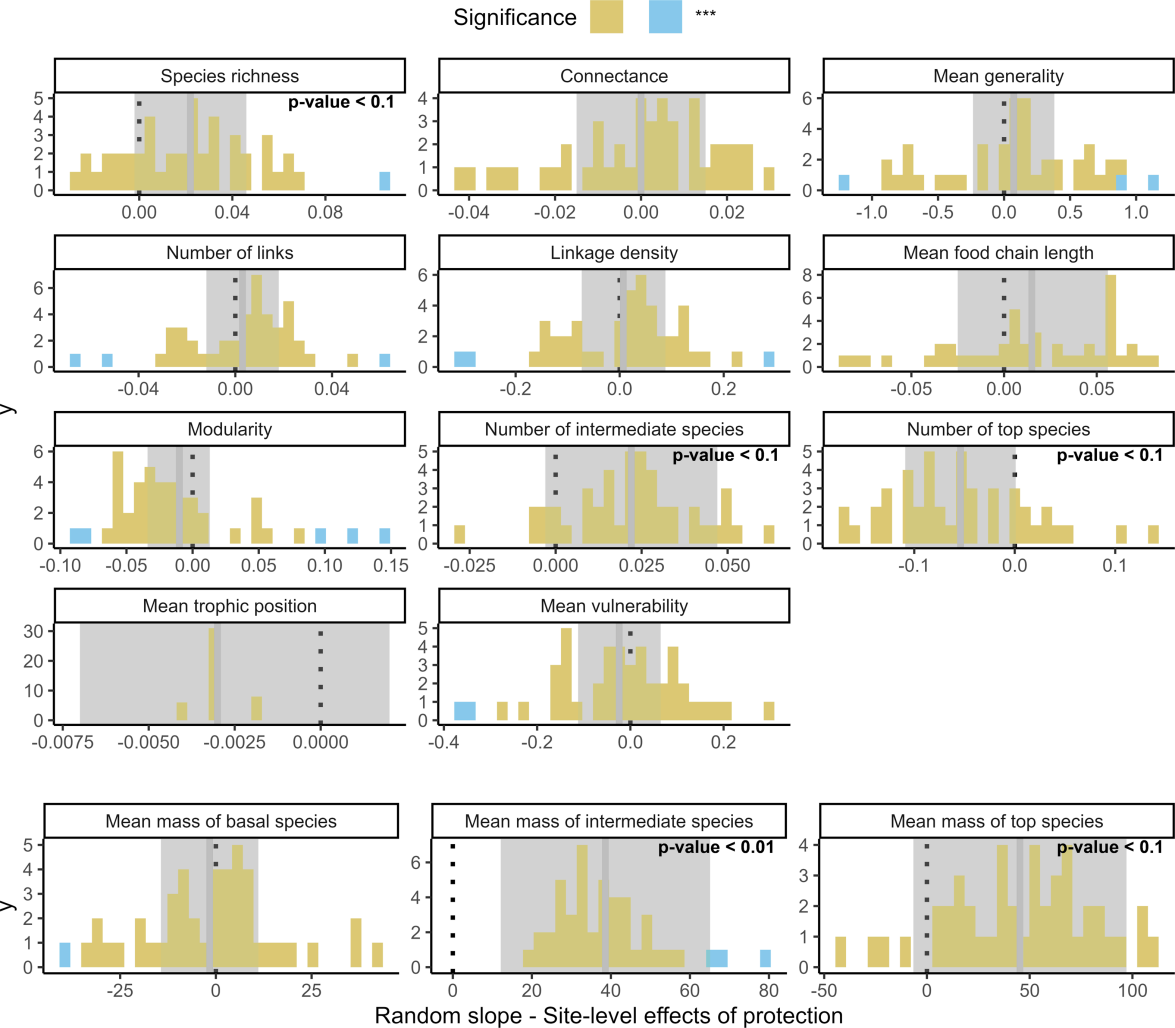
Effect of protection on food web metrics in European protected areas. Results from the GAMMs relating food web metrics to protection status. Grey thick bars and shaded grey area represent β the overall effect of protection on food web metrics across all PA networks and 95% CI. Histograms show the distributions of the 45 PA network-level random slopes $\beta_{i}$ which correspond to the difference in food web metric between protected and non-protected food webs. The colours in the histogram represent significance of $\beta_{i}$ relative to β based on 95% CI. Yellow bars show non-significant differences while blue bars show that PA networks show significant deviation. Positive differences $\beta_{i}$ (positive value on the *x*-axis) indicate PA networks where the effect of protection resulted in a higher food web metric inside PAs than outside. Fraction of omnivory and number of basal species were left out because no variation was observed across $\beta_{i}$s for these two metrics (this can be visualized in the electronic supplementary material, figure S1*c*).

This yielded 16 β coefficients for the overall difference in network metrics across all PA networks as well as 45 × 16 PA network-level $\beta_{i}$ coefficients from the random slopes of the protection effect on each of the 16 food web metrics. We also extracted the 95% confidence interval (CI) around each $\beta_{i}$ using the *mixedup* R package [51] and considered them significantly different from the overall β if the 95% CI did not overlap β (blue bars in the histograms of figure 2).

#### (ii) Drivers of protection effectiveness across protected area networks

To assess the role of environmental conditions in modulating $\beta_{i}$ each PA network was characterized by 15 environmental drivers (more details in the electronic supplementary material):

(i) *PA characteristics* including the average date of designation of its constituting PAs, average protection level, a coefficient of aggregation of its PAs (inverse of fragmentation), the proportion of forested areas inside the reserves [50] and the proportion of PAs managed for birds or PAs under the Natura 2000 bird directive and the Ramsar sites for wetlands [17];
(ii) *PA network-level characteristics* including average elevation [47], slope [47], remoteness [52] and land cover diversity based on CORINE Landcover [50];
(iii) *characteristics of the surrounding landscape* including the mean human density [53], and the mean proportion of urban, agricultural and forested area; and
(iv) the *difference with surrounding landscape:* the difference between the mean values inside and outside for the proportion of agricultural, forested and urban areas as well as human density.

We then quantified which environmental drivers best explained food web metric differences $\beta_{i}$ using multiple linear regressions, controlling for the proportion of protected grid cells across PA networks equation (2.2):


(2.2) $$\begin{array}{l} \beta_{i}\;∼\;proportion\;of\;protected\;grid\;cells+\;\sum PA\;network\;characteristics+\;\sum PA\;characteristics+\;\;\sum \;surrounding\;landscape\;characteristics\;+\;\sum differences\;in\;environmental\;conditions\;\left(inside\;–\;outside\right). \end{array}$$

Given our relatively small number of PA networks (*n* = 45), we used variable selection based on Bayesian information criterion (BIC=ln(n)k−2ln(L) with *k* = number of parameters, *L* = likelihood of the model, *n* = sample size). We extracted model coefficients, standard errors, *p*-values and adjusted *R*² to assess the performance of the linear models and the direction and magnitude of the effect of each environmental variable on the differences in food web metrics $\beta_{i}$. CIs (at 95%) for visualization were extracted from the multiple regression visualization tool *visreg* [54].

#### (iii) Sensitivity analysis

To assess the extent to which results were influenced by our inclusion of partially protected grid cells as ‘protected communities’ we additionally conducted a sensitivity analysis, varying our coverage threshold for defining protected grid cells (more details in the electronic supplementary material). When enforcing a 50 and 90% overlap of protected cells with a PA, the number of PA networks included in the analyses was reduced from 45 to 34 and 16, respectively. We ran the first stage of analysis and compared significance of protection effects on food web structure across these different datasets. All analyses were performed on R v. 4.3.0 [55].

## 3. Results and discussion

### (a) Mixed effects of protected areas on food webs

Our dataset comprised 233 771 survey events reporting 376 556 observational records of 509 bird species across 45 European PA networks, composed of an average of seven (s.d. = 6, min = 1, max = 123) PAs and their surrounding areas (figure 1; see the electronic supplementary material, Data 2 for list of PA networks and their characteristics). Conditions inside PAs were generally more representative of natural environments than their surroundings, with wider forest cover, less area converted to agriculture and smaller human density (figure 1).

Our findings suggest that the establishment of PAs has had an overall positive effect on species richness and the mean body mass of intermediate and top species in avian food webs across Europe (figure 2). It has also prompted a reduction in the number of local top species in favour of larger higher trophic top species, subsuming intermediate consumers (figure 2). For the remaining food web properties studied here, we found no overall significant effect of protection. Even though the overall differences in food web metric were mostly neutral, we observed a variety of outcomes across European PAs. Although not all significant, a substantial number of PAs did show an increase in MFCL (66% of PA networks), number of intermediate species (85% of PA networks) and mean vulnerability (62% of PA networks) compared to their non-protected surroundings (figure 2).

In empirical and theoretical food webs, longer MFCL and more intermediate species are typically associated with higher productivity of habitats [31] or larger and better connected PA networks [56], i.e. habitats that can host more species (but see [57]). Intermediate species act as both resources and consumers, linking top and basal species, and thus creating indirect interactions and facilitating the flow of energy from lower to higher trophic levels [5]. It must be noted, however, that more connected ecological networks can also facilitate the spread of disturbances across species in the ecosystem [32]. Better connectivity across trophic levels might thus not always be desirable for community persistence, but when these changes are accompanied by longer food chains prompted by the presence of higher top predators, as found here, they can have stabilizing effects on ecosystems [58].

The signs of the overall differences in food web metrics were robust to reduction in dataset size and edge effects for 11 out of our 16 metrics under the 50% coverage criterion with 34 PA networks (electronic supplementary material, figure S1*a* and table S1), although significance levels changed. As expected, however, the sharp decrease in the number of PA networks included in the analysis for the 90% overlap dataset had a stronger impact on the effect of protection on food web metrics, and only 7 out of the 16 metrics had consistent sign of effects with the main analysis (electronic supplementary material, figure S1*b*). We kept the largest sample size possible (45 PA networks based on the centre criteria for assigning protection status) to ensure a diverse array of PA networks to maximize robustness and generalizability of results.

### (b) Larger top species promote longer and connected food chains inside protected areas

Modularity and number of top species were found to be on average smaller within PAs compared to the outside across 69% (versus 24% larger) and 73% (versus 9% larger) of PA networks, respectively (figure 2). In this study, species sitting at the top of local (grid cell-level) food webs might not necessarily be top predators in the metaweb. The absence of metaweb-level top consumers prompts a shift of originally intermediate consumers to top positions. PAs holding significantly less top species (min = 0, max = 8, mean = 5.5) also displayed longer MFCLs (min = 2.0, max = 5.4, mean = 3.4) and higher mean trophic position (min = 1.1, max = 1.8, mean = 1.3) than the surrounding non-PAs (Pearson *r* = −0.60, *p*‐value < 0.001 for MFCL versus number of top species). This suggests that higher positioned top predators link many food chains together by predating on various smaller predators that occupy top positions in their respective chains when higher metaweb top predators are locally absent. This was the case for example of the northern goshawk (*Accipiter gentilis*), the Spanish imperial eagle (*Aquila adalberti*) and the rough legged buzzard (*Buteo lagopus*) who were present in the longer food chains and predated on other raptors that became local top predators in shorter food chains in their absence. This in turn reduces modularity by linking different food chains through smaller or lower trophic-level predators that do not overlap in their diets.

To further explore this hypothesis, the role of top species in PAs versus non-PAs and thus find a mechanistic link between a loss of modularity and the number of top species within PAs, we measured the difference in body mass of top species between protected and non-protected cells across PA networks. Across all PA networks examined, the mean body mass of intermediate species was significantly higher (*p*‐value < 0.01) inside PAs than outside by an average of around 40 g (figure 2). Similarly, the mean body mass of top predators was marginally significantly higher (*p*‐value < 0.1) inside PAs than outside by an average of around 50 g and higher inside in 91% (i.e. 41) of the PA networks.

In addition, body mass of the top species was positively correlated with MFCL across PA networks (Pearson correlation *=* 0.23, *p*‐value < 0.001). This shows the capacity of PAs at harbouring larger species that are important for ecosystem stability and functions [23,59].

Together these findings indicate that PAs increase food web complexity by harbouring larger top predators capable of linking ecosystem compartments and making food webs longer and more pyramidal, thus promoting functioning and stability of food webs as well as associated ecosystem services [60].

### (c) Environmental context and management goals drive protected area effects

To unveil the drivers behind the large variability in the differences of food web metrics inside and outside PAs $\beta_{i}$ across PA networks, we assessed the relationships between $\beta_{i}$ and characteristics of PAs, their non-protected surroundings, as well as their differences using linear regressions. Stepwise variable selection based on BIC revealed significant drivers of change for 9 of the 13 food web metrics and 2 out of 3 body size metrics. The effect of PAs on MFCL, number of top and intermediate species and modularity were well explained by a subset of environmental drivers (respective adjusted *R*² = 0.37, 0.33, 0.28 and 0.22; electronic supplementary material, figure S2, tables S2 and S3). By contrast, the mean body mass of intermediate and top species, number of links, species richness, mean vulnerability, linkage density and connectance showed less consistent relationships (adjusted *R*² between 0.12 and 0.07; electronic supplementary material, figure S2, tables S2 and S3).

In particular, elevation, slope (i.e. uphill terrain gradient) and habitat heterogeneity of PA networks constitute strong drivers of the effectiveness of protection. Our results suggest that MFCL ($scaled\;\bar{\beta }$ = 0.030, s.e. = 0.0069, *p* < 0.001, d.f. = 40; electronic supplementary material, figure S3b) and the number of intermediate species ($scaled\;\bar{\beta }$ = 0.011, s.e. = 0.0037, *p* < 0.01, d.f. = 40; electronic supplementary material, figure S3c) in food chains of protected remote locations (i.e. high elevation) increases, while the number of top predator species decreases ($scaled\;\bar{\beta }$ = −0.0426, s.e. = 0.0114, *p* < 0.001, d.f. = 40; electronic supplementary material, figure S3d), relative to their surroundings. Mean slope across the PA network, on the other hand, had a significantly positive effect on differences in linkage density and connectance ($scaled\;\bar{\beta }$ = 0.0477, s.e. = 0.0231, *p* < 0.05, d.f. = 41; $\bar{\beta }$ = 0.007, s.e. = 0.0034, *p* < 0.05, d.f. = 41; electronic supplementary material, figure S3*a*) and a negative one on food web modularity ($scaled\;\bar{\beta }$ = −0.0277, s.e. = 0.0093, *p* < 0.01, d.f. = 40; electronic supplementary material, figure S3b), between protected and non-PAs. These results suggest that the well-known remoteness and difficulty of access to protected ecosystems can actually enhance their food web structures compared to surroundings and highlight the importance of protecting alpine ecosystems [61].

**Figure 3.**
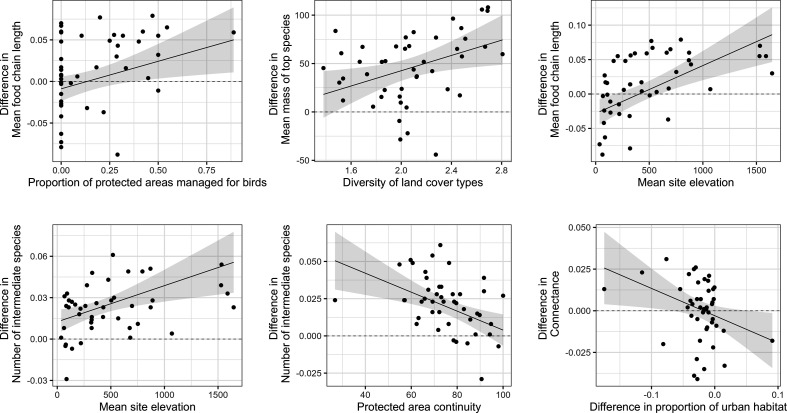
Environmental drivers of the effects of protection on avian food webs across Europe. Partial effects of proportion of PAs managed for birds, elevation, protected area connectivity, diversity of land cover types and the difference in proportion of urban habitat inside versus outside of the PA (among other environmental drivers—see the electronic supplementary material, figure S2) on the differences $\beta_{i}$ in food web metrics and species body size. Points on scatter plots represent the random slope coefficients $\beta_{i}$ of the PA network-level effect of protection on food web metrics (*n* = 45 PA networks, average 60 grid map cells per PA network). Positive differences indicate higher food web metrics inside than outside PAs. Lines and shaded area around them show the partial linear regression with 95% confidence interval (see the electronic supplementary material, figures S3a-d for all scatter plots and tables S2–S3 for the full scaled and non-scaled results, respectively).

Lastly, habitat heterogeneity, assessed here as the diversity of land cover types, had a positive effect on the body mass of top predator species inside PAs ($scaled\;\bar{\beta }$ = 14.47, s.e. = 5.77, *p* < 0.05, d.f. = 42; figure 3), highlighting the importance of a variety of habitats to sustain larger species at higher trophic levels [62] capable of regulating food webs from the top down [23].

Furthermore, PA networks with a higher proportion of PAs specifically managed for birds (Natura 2000 bird directive sites and Ramsar sites for wetlands) hosted PAs with longer MFCL ($scaled\;\bar{\beta }$ = 0.014, s.e. = 0.0055, *p* < 0.05, d.f. = 40; electronic supplementary material, figure S3*b*), less top species ($scaled\;\bar{\beta }$ = −0.024, s.e. = 0.0089, *p* < 0.05, d.f. = 40; electronic supplementary material, figure S3d) and less vulnerable species ($scaled\;\bar{\beta }$ = −0.052, s.e. = 0.02, *p* < 0.05, d.f. = 41; electronic supplementary material, figure S3b) than their surroundings. This reveals that the relatively small positive effects of PAs on avian food webs (figure 2) are partially modulated by the purpose for which PAs are designated. Natura 2000 areas of protection have been created with the aim to fulfil European Directive 2009/147/EC of 30 November 2009 for the conservation of wild bird populations [63], to preserve, maintain or restore a sufficient diversity and area of habitats for the conservation of all species of birds, with special conservation measures for the habitats of certain species to ensure their survival and reproduction in their area of distribution. The Ramsar protocol aims to promote the conservation of wetlands and waterfowls [64]. Our results suggest that both Natura 2000 and Ramsar designations are fundamental not only to protect bird diversity but also to the conservation of biotic interactions and the structure of the ecological networks they shape.

### (d) The influence of anthropogenic disturbances

Anthropogenic disturbances had a strong effect on the effectiveness of PAs in safeguarding avian food webs. PA networks with large proportions of agricultural area outside of the PA correlated positively with the difference in the number of intermediate species ($\bar{\beta }$ = 0.082 s.e. = 0.03, *p* < 0.01, d.f. = 37; electronic supplementary material, figure S3c), suggesting that a variety of habitats, even if anthropogenic, can contribute to species richness in intermediate trophic levels. These results suggest that establishing PAs in anthropogenic landscapes can successfully enhance avifaunal diversity.

Higher human density inside PAs compared with the outside made food webs more modular ($\bar{\beta }$ = 0.0193, s.e. = 0.007, *p* < 0.01, d.f. = 41; electronic supplementary material, figure S3a), very possibly owing to its negative influence on the top trophic level exerted through an increase in the number of local top species ($\bar{\beta }$ = 0.0265, s.e. = 0.0092, *p* < 0.01, d.f. = 40; figure 3), reflecting the absence of metaweb top-predators capable of connecting modules across the food web. Arguably a more pervasive effect than human density *per se* was that observed for the proportion of habitat that has been converted to urban development, which influenced a suite of network properties. PAs with larger urban areas than their surroundings, even if having more species ($\bar{\beta }$ = 0.0108, s.e. = 0.0041, *p* < 0.05, d.f. = 42; electronic supplementary material, figure S3a), harboured less connected food webs (connectance: $\bar{\beta }$ = −0.0061, s.e. = 0.0026, *p* < 0.05, d.f. = 41; linkage density: $\bar{\beta }$ = −0.0413, s.e. = 0.0173, *p* < 0.05, d.f. = 41; electronic supplementary material, figure S3a) with shorter MFCLs ($\bar{\beta }$ = −0.0111, s.e. = 0.0056, *p* < 0.1, d.f. = 40; figure 3).

### (e) Preserving biodiversity beyond species richness

We have shown how the composition and topology of the avian food webs differ between PAs and non-PAs across diverse biogeographical regions of Europe and identified potential drivers of these differences. The geographical (i.e. elevation and slope) and environmental context (i.e. diversity of land cover types) of locations at which PAs are established emerged as predictors of the effects of PAs on a suite of food web metrics. In addition, anthropogenic disturbances around and inside PAs also affect MFCL, number of feeding links and number of top species. Finally, PAs specifically managed for the protection of birds harbour avian food webs with longer food chain lengths, less top predator species but occupying higher trophic levels and with less vulnerable species in general.

Even though we did find a positive effect of PAs on species richness, we were unable to tie these differences to a particular driver, except for a small effect of urban habitat (see full results in the electronic supplementary material, figure S3 and tables S2, S3. By contrast, many abiotic features emerged as potential drivers of changes in biodiversity when ecological interactions, and their structure, are considered. Thus, our results demonstrate the importance of moving beyond species to develop more accurate and multifaceted biodiversity assessments and highlight the potential added value of a holistic approach to assessing the effect of conservation strategies.

### (f) Mixed effect of protected areas on food web metrics

At the continental scale, we found that European PAs only significantly influence a relatively small set of network metrics. This suggests either a weak effect of protection on food webs and/or potential spillover effects between PAs and non-PAs. The prevalence of negligible differences in food web metrics between PAs and non-PAs aligns with previous findings on the mixed effect of PAs on waterbird population trends [17]. This was the case even for metrics like mean generality and vulnerability related to species’ specialization contrary to Cazalis *et al*. [15]. Such lack of difference in food web structure between protected and non-protected communities could arise from different mechanisms. First, in Europe, where most of the landscape has been heavily anthropized, most PAs allow for some level of anthropogenic activities (e.g. International Union for Conservation of Nature category V) and are often designated for multiple functions, including recreational purposes [65] or the historical protection of game species [66]. Thus, thinking of PAs as a single disturbance gradient is reductive. They can act on many different facets of our landscapes, from reducing land use intensification, human density to conserving cultural values. This multifunction and disparity across PAs could be responsible for the blurred signal in terms of protection outcome. Second, species dispersal across landscapes and the resulting rescue or spillover effects [67]—where, through the dispersal of species, higher productivity habitats maintain disturbed habitats—have been shown to promote biodiversity at regional scales in simulated heterogeneous landscapes, even in the presence of highly disturbed habitats [67]. Third, on the contrary, more homogeneous landscapes, e.g. either remote and relatively untouched PA networks or highly anthropized landscapes with high levels of human activities within PAs, might display similar food web structure inside and outside PAs, regardless of whether some land is put aside for protection. In these cases, protection status may be a simple label resulting in limited tangible reductions in anthropogenic disturbances, as the landscape was already largely undisturbed prior—or on the contrary remains highly disturbed despite—PA establishment. Finally, despite evidence that trophic network structure varies along environmental gradients [7,28,49], it has also been argued that the structure of networks is relatively consistent across space and biomes [68] (i.e. species and/or interactions are replaced but structural properties remain the same). This might make differences in food web structure between PAs and their surroundings harder to detect.

### (g) The importance of protected area management practices and landscape features

Similarly to Wauchope *et al*. [17] we found that PAs managed for birds (PAs under the Natura 2000 bird directive and the Ramsar sites for wetlands) are more effective at hosting food webs with longer MFCLs featuring a smaller number of, usually bigger, top species that prey upon a larger intermediate guild. Those two conservation strategies that date back to the Bern convention in 1979 when the Natura 2000 sites and the Birds Directive were created to ‘support the provision of a coherent EU-wide ecological network of sites’ [66] seem to significantly affect some aspects of food webs, attracting larger bodied top predators and promoting longer food chains. Thus, PAs with specific management goals seem to yield better outcomes for communities [16,17].

Anthropogenic impact outside PAs, notably the detrimental effect of human density and urban habitat surrounding PAs affects MFCL, number of top species and mean number of feeding links. Both human density and the proportion of urban habitat influence food web structure by decreasing connectivity and reducing food chain length. Increased anthropogenic impact also increased the number of local top species at the expense of fewer but higher trophic top species, hinting at the disappearance of top predators that preyed upon these ‘local top species’ (intermediate species at the metaweb-level). As highlighted by previous theoretical work on the design of PAs [69], the integrity of the landscape outside reserves can promote species persistence inside reserves. This importance of surrounding habitats is increasingly considered in the design of reserves, with the integration of buffer zones and dispersal corridors around PAs [11].

At higher elevations, MFCLs inside PAs are longer because of a larger intermediate consumer guild sitting below fewer top predators. PAs in locations with steeper slopes display more connected and less modular food webs, whereas a diversity of habitats promotes the presence of larger top predators. These beneficial effects are further enhanced by the management goals of PAs. Areas of high elevation and slope in our study are mostly occupied by alpine ecosystems. These ecosystems are unique and have been heavily impacted by different drivers of global anthropogenic change such as climate warming [61] or recent encroachment owing to land abandonment [70], which have had particularly important effects on food webs such as reduction on body size structure and decreasing specialization. By revealing the potentially strong positive impacts that PAs can have on the structure of avian food webs in alpine ecosystems, our results, together with these previous findings, highlight the key importance of prioritizing these ecosystems in future conservation efforts.

The importance of the diversity of land cover types for conserving larger top predators is interesting and can be linked to their vulnerability to habitat fragmentation [71] and their natural occupation of larger areas [56].

## 4. Limitations

Although our original aim was to make our study as global as possible in terms of species, geographical coverage and types of PAs, scarce biodiversity survey data caused our study to be biased towards specific European bioregions (Atlantic, Alpine and Mediterranean) and taxonomic groups (bird species only). Indeed, 94.5% of the European occurrence records downloaded from GBIF were for avian species, with only 4.1% of mammals and 0.9% amphibians and 0.5% reptiles. This highlights the important limitations caused by this well-known taxonomical bias towards avian taxa in citizen science data [72]. Given that many PAs do not have as primary focus the protection of birds, but aim at preserving other species or aspects of ecosystems [73], it would be relevant to repeat this analysis on the full food web of tetrapods, perhaps using broader range maps (similarly to e.g. [49]) instead of occurrence data, in order to compare results. Another limiting factor precluding a more comprehensive analysis of food webs in PAs across the globe is the availability of ecological interactions data. Even though several ecological networks have been characterized across the globe (e.g. web of life *https://www.web-of-life.es/,* GloBI https://www.globalbioticinteractions.org/ or Mangal https://mangal.io/), to our knowledge, the Tetra-EU [43] European tetrapod food web is the only well-resolved continental-scale ecological network characterized to date. Filling this data gap is fundamental to develop a comprehensive assessment and understanding of the effectiveness of PAs at preserving a fundamental aspect of biodiversity: the structure of ecological interactions.

In this study, we assumed that two species would interact as long as they co-occur and a trophic link in the European metaweb exists between them throughout the breeding period. However, species interactions are known to vary across environmental gradients [7,49], time and spatial scale [74]. This can be owing to several factors including species turnover, changes in species abundances across habitats or interaction rewiring [75]. Given the spatial extent of our study which encompasses several biogeographical regions (Atlantic, Continental, Boreal, Alpine and Mediterranean) with very different and unique environmental features, where in some cases there is a strong species overlap but also replacement, which can impact food web structure [68], it is reasonable to believe that our assumption of constant interaction through space and time is a simplification which could bias our results.

The relatively small proportion of positive outcomes found here does not suggest that protection is ineffective. First, here, we only consider avian species, and our results ignore a whole portion of realized food webs. Second, our measure of the difference in food web metrics does not consider the absolute value in food web metric. Thus, regions with consistently ‘good’ or ‘bad’ food web metrics (see paragraph below) will both display neutral difference. These nuances are important and are the reasons why we refrain from talking about PA performance or effectiveness. We simply aim to uncover how food web structure varies spatially with respect to PAs. Furthermore, quantifying the effect of protection would be much improved with a before after comparison (e.g. [17]), complementing the spatial comparison. With more resolved data, an analysis of how food web metric varies with distance to PA boundary would permit us to explore the change in absolute food web metrics rather than their difference inside and outside with respect to protection. This would allow us to quantify spillover effects from PAs, or the effects of having buffer areas around reserves [11].

Finally, we believe that a strong limitation to designing conservation strategies in the context of holistic ecosystems protection is the lack of clear guidelines. Indeed, it is not straightforward to identify which metrics are to be preserved. In the context of trophic networks, depending on the conservation goal and disturbance context, we might wish to conserve rare trophic specialists or keystones species [76] or increase stability through enhancing food chain length, modularity [77,78] or complexity. In addition, integration of spatial considerations such as connectivity and species dispersal adds an extra layer of complexity [79,80]. Thus, the question of ecological network stability is highly multidimensional, resulting in a strong disconnect between theoretical and empirical studies [81,82]. This has led to limited consensus on the impact of disturbances on ecological stability [81] potentially limiting their integration into conservation. Nonetheless, ecological interactions are a fundamental missing piece in the puzzle of preserving ecosystems and their services under current threats [83] and should thus be further incorporated into conservation efforts.

## 5. Conclusions

We find clear effect of protection on intermediate and top species, with PAs hosting larger intermediate species and predators which are known to play crucial roles in connecting ecosystems and species. On other aspects of food web structure, however, our results suggest mixed effects of protection. Notably, we highlight that protection outcome is highly context dependant, with no single PA network type managing to enhance all aspect of food web complexity and structure. Our results support the creation of reserves with favourable context and well-set management goals, following the positive effects of the proportion of PAs managed for birds and the importance of landcover type and anthropogenic pressure inside and outside reserves. Had we only considered species richness in our study, we would have concluded that only urbanization had a minor influence on the effects of PAs on biodiversity. We provide insights into the benefits of evaluating the outcome of protection from a community perspective and contribute to the growing realization of the importance of surrounding landscapes for PAs.

## Data Availability

Data produced for intermediate steps in the analyses (supplementary Data 1–3) are available at: https://figshare.com/s/fa2913a16c614dfe3b07, doi: 10.6084/m9.figshare.24645543. All data were extracted/downloaded from open sources. Biodiversity records are accessible from the GBIF at [41]. Dois for the GBIF downloads are available in the electronic supplementary material, Methods, table 1.eBird version of May 2022 for all European countries is available at [42]. Land cover data are available from [50]. The TETRA-EU 1.0 dataset of species interactions and biogeographical region shapefile are available at [43]. The World Database on Protected Areas (WDPA) March 2022 version is available at [44]. Elevation from the Shuttle Radar Topography Mission (SRTM) is available at [47] under 'Elevation' Remoteness data available at [52] under 'Travel time to cities'. Human density data for 2015 came from the Gridded Population of the World dataset, version 4.11 (GPWv4.11, UN WPP-Adjusted Population Density, v4.11, 2015) available at [53]. Computer code for all the analyses (including data processing) presented in this study is available on Figshare [84]. Computer code is also available on GitHub [85].
